# Identifying the Best-Fitting Factor Structure of the Experience of Close Relations - Revised in a Scandinavian Example

**DOI:** 10.1371/journal.pone.0137218

**Published:** 2015-09-02

**Authors:** Barbara Hoff Esbjørn, Sonja Breinholst, Janni Niclasen, Louise Fabritius Skovgaard, Katrine Lange, Marie Louise Reinholdt-Dunne

**Affiliations:** Department of Psychology, University of Copenhagen, Copenhagen, Denmark; Tilburg University, NETHERLANDS

## Abstract

The aim of this study was to enhance the understanding of cultural and sample differences in the assessment of attachment by examining the factor structure of the Experiences in Close Relationships-Revised (ECR-R). The ECR-R is a self-report measure of adult romantic attachment dimensions. The present study used a Danish sample with the purpose of addressing limitations in previous studies, such as the lack of diversity in cultural background, restricted sample characteristics, and poorly fitting structure models. Participants consisted of 253 parents of children between the ages of 7 and 12 years, 53% being mothers. The parents completed the paper version of the questionnaire. Confirmatory Factor Analyses were carried out to determine whether theoretically and empirically established models including one and two factors would also provide adequate fits in a Danish sample. A previous study using the original ECR suggested that Scandinavian samples may best be described using a five-factor solution. Our results indicated that the one- and two-factor models of the ECR-R did not fit the data well. Exploratory Factor Analysis revealed a five-factor model. Our study provides evidence that further investigation is needed to establish which model may provide the best model fit in the Scandinavian countries.

## Introduction

### Measuring attachment: A historic overview

Over five decades ago Bowlby developed his attachment theory and thereby presented one of the most dominant frameworks for understanding early socio-emotional development [1; 2; 3]. This has led to an extensive study of how attachment bonds between a parent and an off-spring could be adequately operationalized and measured regardless of whether the offspring was a child, an adolescents or an adult [4]. In relation to adults, Hazan and Shaver’s [5] influential work suggests that attachment theory may also be applied to studies of romantic relationships [6]. Naturally, a proper operationalization of the construct with reliable research instruments continues to be pivotal, perhaps even more so as attachment research is conducted in various cultures and settings, for instance to describe the romantic relationships in clinical samples. In response to this issue, Brennan, Clark and Shaver developed the Experiences of Close Relationships (ECR) [7], based on items from a pool of existing self-report measures of adult romantic attachment. They analyzed 323 items administered to 1086 undergraduate students. This work resulted in the creation of two factors: Avoidance and Anxiety. The Avoidance subscale characterized individuals who avoided intimacy or felt discomfort with closeness, whereas the Anxiety subscale characterized individuals who were afraid of being rejected or abandoned. Each of these subscales consisted of the 18 highest loading items from the original item pool. It has been suggested, that adults scoring high on either of these two dimensions have an insecure attachment [7].

Research on the psychometric properties of the ECR has revealed problems regarding the precise measurement in particular the ‘secure’ end of each attachment dimension [8; 9; 10]. One of these problems pertains to the scoring of the scales. When scoring the scale, individual’s responses may be calculated as an average of the two scales. Although attachment researchers often opt for reporting the two dimensions separately, individual’s responses may also be calculated as an average of the two scales. This total average may be skewed towards security as higher scores on only one scale would be masked by lower scores on the other scale [9]. Another potential problem with the ECR is related to its factor structure in different cultures. Although most studies confirm a two-factor structure, a Scandinavian study proposed a five-factor structure as the best model fit for a Norwegian sample [11]. This latter finding may be a result of weaknesses of the ECR, which could be eliminated in the creation of a revision of the ECR, or they could be a result of cultural differences.

### Experiences in Close Relations—Revised

In order to improve the accuracy and reliability of the questionnaire, Fraley, Waller and Brennan developed the Experience of Close Relations—Revised (ECR-R) [9]. The ECR-R is now the most frequently used self-report measure of adult romantic attachment dimensions [12; 13]. It was developed by reanalyzing the original comprehensive 323 items using factor analytic and item-response paradigms. The application of Item Response Theory, allowed Fraley et al. [9] to capture a more precise relationship between an individual’s item responses and underlying latent traits. This resulted in a new 36 item version of the questionnaire with ratings on a 7-point Likert scale. The items continued to be distributed on the two scales; Avoidance and Anxiety. The ECR-R encompasses seven items from the original ECR’s 18 avoidance items and 13 items of the original ECR’s 18 anxiety items. In the following studies of the psychometric properties of the ECR-R, strong evidence was found for an increased measurement precision [9; 13].

Overall, the psychometric properties of the ECR-R have been tested in nine studies [8; 10; 13; 14; 15; 16; 17; 18; 19]. The studies highlight three limitations that have, however, yet to be overcome. First, although theory suggests a two-factor model, a significant number of the conducted studies have not been able to fully confirm this model [8; 10; 14; 19]. Secondly, to our knowledge, there are no Scandinavian studies on the ECR-R. This affects the cross-cultural validity and applicability of the questionnaire. As a Scandinavian study of the ECR suggested possible cultural differences, a factor analysis of the ECR-R in a Scandinavian sample is warranted. The third limitation is concerned with sampling characteristics. In six out of the aforementioned nine studies, undergraduate students were recruited as respondents. As the ECR-R measures romantic relationships, it is likely that young adults in their first relationship will differ from older adults, and parents, who have been in longer lasting relationships and under the strain of parenthood.

The aim of the present study has therefore been to address these limitations and examine the ECR-R in a Scandinavian country in order to illuminate potential cultural and sampling differences in the assessment of attachment. We examined the factor structure of the ECR-R in a Danish sample of adults primarily in their thirties and forties. These adults were parents of children aged 7–12 years. Based on the only previous study which has included adults in the Scandinavian countries, we hypothesized that the theoretically suggested one- and two-factor structures would not adequately fit our data. Factors such as age, experiences in close romantic relationships, life experience and parenthood may contribute to the lack of fit to the theorized one- and two-factor models. We have therefore also explored our data hypothesizing that our sample will show tendencies similar to those of the previous Scandinavian study, which yielded a five-factor solution.

## Methods

### Participants and procedure

The sample consisted of 253 parents of children aged 7 to 12 years. The majority of the participants (n = 207) were parents of clinically anxious children. This group consisted of 109 mothers and 98 fathers. The remaining participants (n = 46) were parents of typically developing children. This group consisted of 25 mothers and 21 fathers. All parents completed paper versions of the ECR-R at home prior to attending an individual appointment at a university clinic with their child as part of a larger study aimed at understanding childhood anxiety. The parents did not receive any financial remuneration. Of the 253 parental ratings of the ECR-R, a total of 134 (53%) were obtained from mothers.

The study complied with all ethical standards regarding research conducted on human samples, and was approved by the Institutional Ethical Review Board, University of Copenhagen, Department of Psychology. Written informed consent was obtained from all participating families. The ECR-R was translated into Danish using a translation/back-translation procedure [20] with items placed in a random order as suggested by Fraley et al. [9]. The original questionnaire was translated by a staff member, following which, a second staff member, who had not read the original questionnaire, performed the back-translation.

### Statistical analyses

#### Missing data

Initially, the sample consisted of 288 participants. Of these, 34 had missing values on all 36 ECR-R items and were excluded from the analyses. One further person had missing values on 26 items and was also excluded. This left 253 ratings for the analyses. None of these had more than two missing values on the 36 items. Person mean substitution was used to replace missing values, i.e., a mean of the ECR-R items was calculated for each person and this value was used to replace the missing value.

#### Factor analyses

We applied Confirmatory Factor Analysis (CFA) to test the one- or two-factor structure of the ECR-R in our Danish sample. All CFAs were carried out in the statistical package Mplus version 6. We used the WLSMV estimator, designed for use with small and medium sample sizes [21]. Because most items had a skewed distribution, the data were treated on a categorical level. Model fits were evaluated by Chi Square Test of Model Fit where 0 indicates a perfect fit. Three indicators of fit were computed. The Steiger-Lind Root Mean Square Error of Approximation (RMSEA) < 0.08 indicates an acceptable model fit and < 0.05 a good model fit. For the Tucker-Lewis Fit Index (TLI), and the Bentler Comparative Fit Index (CFI) > 0.90 signifies acceptable fits and > 0.95 signifies good fits [22]. When certain parts of the model did not show acceptable fits, loadings between specific indicators were allowed for on the basis of modification indices if they were considered theoretically meaningful.

Our data were further explored by means of Exploratory Factor Analyses (EFA). The reason for this exploration was the very poor model fits obtained in the CFA for the one- and two-factor models and that a previous study of the original ECR [11] suggested that a five-factor solution may provide the best model fit in Scandinavian samples. The EFA was carried out in the statistical package SPSS version 21. The number of factors retained in the analyses was based on the Kaiser principle, i.e., factors with an initial Eigenvalue > 1 were kept in the analyses and subjected to orthogonal rotation.

## Results

### Descriptions of the sample

Prior to conducting factor analyses, we assessed the demographic characteristics of the participants in each group. Comparisons between groups were conducted using independent samples *t*-test and chi-square. There were no significant differences in age of the parents of clinical children vs. non-clinical children (mean age (SD): 40.3 (4.3) versus 41.4 (5.0) years for mothers; and 42.1 (5.2) versus 43.3 (6.4) years for fathers). The age range was 31–52 years for mothers and 32–60 years for fathers. There were also no significant differences in the percentages of parents who were single versus cohabiting in the two groups (81% vs. 64% of mothers of non-clinical vs. clinical children were cohabiting; as were 81% vs. 70% of fathers). We found a trend (*p* = .053) for non-clinical families to have a higher yearly household income than families with clinical children (mean income equivalent to 500.000–750.000 Danish kroner versus 300.000–500.000 Danish kroner). Fathers and mothers of non-clinical children had a mean educational level equivalent to education at the university bachelor level versus parents of clinical children who had a medium length vocational education. For mothers this difference was significant (Mean (SD): 4.63 (.62) vs. 3.52 (1.3); t = 5.53, df = 39.3; *p* = .000), for fathers it was only a trend (*p* = .066). Despite slight differences, the samples were sufficiently homogeneous to collapse into one sample. Factor analyses were therefore conduction on the total sample.

### Confirmatory Factor Analysis

We examined the two previously applied models by means of CFA. Model 1 was a one-factor first-order model including all 36 items: ‘Total-experiences in close relations’. Model 2 was based on Fraley, Waller, and Brennan’s originally proposed two-factor, first-order model [9].

As shown in Table 1 both models fitted data extremely poorly. The Chi Squares and RMSEAs were unacceptably high, whereas the CFI and TLI were unacceptably low. Inspection of the modification indices revealed that none of the modifications would substantially improve the model fits.

**Table 1:** Model Fits of the One- and Two-factor Models of the ECR-R.

ECR-R	Chi Square	DF	RMSEA (95% CI)	CFI	TLI
	2427	594	0.110 (0.106–0.115)	0.870	0.862
	2407	593	0.110 (0.105–0.115)	0.871	0.863

Note: DF = Degrees of Freedom. RMSEA = Root Mean Square Error of Approximation. CI = confidence interval. CFI = Comparative Fit Index. TLI = Tucker-Lewis Fit Index.

### Exploratory Factor Analyses

Five factors had Eigenvalues > 1. The five-factor model explained 59.5% of the variance, and was chosen on the grounds of the previous study in a Scandinavian population [11] and attachment theory [4]. See Table 2. Small factor loadings between +/- 0.35 are omitted. Items which clustered on the same factors suggest that Factor 1 depicts behavior associated with being Independent Avoidant, Factor 2 with being Anxious, Factor 3 with being Counter-dependent Avoidant, Factor 4 with being Angry-Preoccupied, and Factor 5 with experiencing Anxious Low Self-regard.

**Table 2:** Principal Component Analysis with Varimax rotation for ECR-R (N = 253).

	Factors				
	1	2	3	4	5
5(R). It’s easy for me to be affectionate with my partner.	**0.51**		-0.46		
7(R). I talk things over with my partner.	**0.73**				
8(R). It helps to turn to my romantic partner in times of need.	**0.71**				
11(R). I usually discuss my problems and concerns with my partner.	**-0.77**				
13(R). I am very comfortable being close to romantic partners.	**0.61**		-0.47		
14(R). I find it relatively easy to get close to my partner.	**0.72**				
31(R). I tell my partner just about everything.	**0.77**				
32(R). My partner really understands me and my needs.	**0.68**				
33(R). It’s not difficult for me to get close to my partner.	**0.66**				
35(R). I feel comfortable sharing my private thoughts and feelings with my partner.	**0.54**		-0.42		
2(R). I do not often worry about being abandoned.		**-0.68**			
6. When my partner is out of sight, I worry that he or she might become interested in someone else.		**0.54**		0.49	
12. I worry a lot about my relationships.		**0.59**			
15(R). I feel comfortable depending on romantic partners.	0.38	**-0.53**			0.44
18. I worry that I won’t measure up to other people.		**0.42**			
28(R). I rarely worry about my partner leaving me.		**-0.63**			
34(R). I find it easy to depend on romantic partners.		**-0.54**	-0.49	-0.36	
3. I prefer not to be too close to romantic partners.			**0.60**		
16. I get uncomfortable when a romantic partner wants to be very close.			**0.68**		
21. I don’t feel comfortable opening up to romantic partners.	-0.52		**0.52**		
24. I find it difficult to allow myself to depend on romantic partners.		0.39	**0.43**	0.39	
27. I am nervous when partners get too close to me.			**0.65**		
29. I prefer not to show a partner how I feel deep down.	-0.38		**0.58**		
36. I’m afraid that once a romantic partner gets to know me, he or she won’t like who I really am.		-0.39	**-0.50**		-0.44
9. My desire to be very close sometimes scares people away.				**-0.53**	
20. It makes me mad that I don’t get the affection and support I need from my partner.				**0.72**	
23. My partner only seems to notice me when I’m angry.				**0.53**	
25. When I show my feelings for romantic partners, I’m afraid they will not feel the same about me.				**0.50**	0.47
26. I find that my partner(s) don’t want to get as close as I would like.				**0.52**	0.40
30. My romantic partner makes me doubt myself.				**0.62**	
1. I often worry that my partner will not want to stay with me.		0.69			**0.41**
4. I worry that my romantic partners won’t care about me as much as I care about them.		0.48		0.36	**0.57**
10. I often worry that my partner doesn’t really love me.		0.49			**0.54**
17. I’m afraid that I will lose my partner’s love.		0.45			**0.66**
19. I often wish that my partner’s feelings for me were as strong as my feelings for him or her.					**0.62**
22. Sometimes romantic partners change their feelings about me for no apparent reason.					**0.60**
Eigenvalue	13.52	3.57	1.75	1.42	1.17
Percentage of explained variance	37.56	9.91	4.85	3.96	3.26

Note: R = reversed item.

Analyses of mean differences between the clinical versus non-clinical groups were conducted for mothers and fathers separately. No significant differences were found. Means and standard deviations for mothers and fathers on each of the five factors are depicted in Table 3.

**Table 3:** Means for the five factors for mothers and fathers (Standard Deviations in Parentheses).

	Mothers	Fathers
Factor	*(n = 134)*	*(n = 119)*
Independent Avoidance	2.19 (1.11)	2.30 (1.12)
Anxious	2.36 (1.20)	2.30 (1.19)
Counter-dependent Avoidance	1.88 (1.09)	1.89 (.97)
Angry-Preoccupied	2.00 (1.08)	1.95 (1.06)
Anxious Low Self-regard	2.33 (1.33)	2.47 (1.25)
Total score	2.15 (.94)	2.17 (.93)

## Discussion

The aim of our study has been to enhance the awareness among researchers within this area of possible cultural and sampling differences in the factor structure of the ECR-R. We aimed at overcoming previous limitations in the literature by employing a sample of respondents (parents) differing to what has been employed in most previous studies (namely undergraduate students). Our study analyzed data derived from adults mainly in their forties, living in a Scandinavian country, and hypothesized that these conditions would affect the fit of the theoretically suggested two-factor model of the ECR-R. The CFA suggested that the theoretically and empirically established one- and two-factor structures did not fit the data from the current sample. Rather, the EFA suggested a five-factor solution. Although our findings do not corroborate most previous research on the ECR-R, they provide an important contribution to the literature due to the different sample characteristics. However, our findings are limited by the lack of independent samples in the CFA and EFA. As splitting the sample in two, would have reduced the sample size and hence the power in our calculations, we applied a dependent sample. We must therefore caution the reader against drawing firm conclusions before additional analyses in independent samples have confirmed our findings.

In the following, we will discuss the implication of our findings in relation to theory, cultural and sample characteristics.

Assessing the distribution of the items, five dimensions emerged: Independent Avoidance, Anxious, Counter-dependent Avoidance, Angry-Preoccupied, and Anxious Low Self-regard. In concordance with the theory, on the ECR and ECR-R, our data suggest that secure attachment is not measured directly. Rather, the lack of insecure responses from an individual will indicate a secure attachment [9]. Furthermore, our dimensions of including avoidance, or anxiety in the attachment relation are in line with attachment theory in general [4]. However, not all scales may be directly understood in the light of attachment theory. In theory, low self-regard would be conceived of as a product of insecure or disorganized attachment rather than as a category in itself. In our study, however, anxious attachment was split in two categories. One depicting the traditional characteristics of anxious attachment, the other depicting anxious low self-regard. The Anxious Low Self-regard factor was associated with higher levels of hopelessness than the factor labeled Anxious. One can speculate that the Anxious Low Self-regard factor may include parents, who doubt themselves in romantic relationships, whereas the Anxious factor includes parents, who are also jealous in their romantic relationships. Furthermore, we found evidence for *two* different dimensions representing avoidant attachment behaviors: one including independent and the other counter-dependent behaviors. The factor of Independent Avoidance lacks expressions of emotional longing. The Counter-dependent Avoidance to a higher degree suggests a longing for close relations but at the same time a fear of being close to romantic partners. A final dimension was Angry-Preoccupied. This dimension has also been described in the literature on the Child Attachment Interview [23]. Based on this interview, an Angry-Preoccupied classification was identified [23] in which participants dwelled on their angry and hostile perceptions in interactions with significant others. Our Angry-Preoccuped factor captures similar views on the close relationship. However, within the structure of the ECR-R, the respondents are not provided with the opportunity to dwell on and elaborate on their views. Future research may therefore consider further examining this dimension of Angry-Preoccupied attachment.

Although, it is premature to aboard the traditional two factor approach to measuring attachment, the nuancing of attachment factors as suggested by our analyses is of potential clinical relevance. E.g. a previous study has reported that children’s anxiety levels were predicted by paternal avoidant behaviors (measured using the ECR-R) and low levels of reflective functioning in mothers [24]. However, it is likely that fathers who are avoidant but longing for a close relationship may be less clear in their signals towards their child, than fathers who are consistently avoidant, and do not seek closeness. Therefore, it may be beneficial for clinicians to understand the finer nuances, in order to directly target parental attachment behaviors maintaining anxiety in the child.

### Possible role of cultural differences and sample characteristics

Turning to the cultural aspects, only one previous study has examined attachment in close relations in a Scandinavian culture [11]. That study questioned the suggested two-factor structure, and proposed a five-factor structure of the ECR. As the ECR and ECR-R do not consist of identical items, we were not able to assess whether the previous model could be confirmed in our sample. However, our EFA suggested a five-factor structure for our data. This is in line with findings by Olssøn and colleagues [11]. We offer two explanations for these findings. 1) They may be due to difficulties in the translation of the questionnaire. This is supported by the fact that some items (2, 9, 11, 15, 28, and 34, 36; see Table 2) loaded negatively on their respective scales, and may be explained by cultural differences in the subjective understanding of the translated items. Therefore, we suggest that the mentioned items should be closely scrutinized and either rephrased or reversed if future studies wish to replicate our study. 2) Our findings arise from cultural differences in norms of interactions. In an attempt to understand Scandinavian individuals’ responses to attachment measures, we have examined the literature on attachment distributions in Denmark. One study of first-time mothers suggested that the distribution of classification patterns was different from that found in most other countries, with more avoidance being reported than has appeared in existing meta-analyses [25]. Although this finding is not directly linked to the number of factors found, it may nevertheless suggest that Danish samples report differently in matters of avoidance on attachment measures than occurs in other cultures.

Finally, sample characteristics such as age, clinical and parental status of participants may have a marked influence on results. Most previous studies, supporting a two-factor structure, have been conducted using undergraduate students. In comparison, our study collected data from adults aged 31–60 years of age and, who had school-aged children. The ECR-R measures attachment in close relationships, and becoming a parent may nuance the understanding of these, yielding a larger variety of attachment-related perceptions of self and others. This may in part explain why we, and two out of three other studies including adults rather than undergraduate students, do not find full support for the two-factor structure [10; 14]. The differences in findings between previous studies and our study may also in part be due to parental status. In the three previous studies using adult samples, it is not reported whether the participants have children. We speculate that having children will also affect the experience of close relationships, and, if so, our findings can therefore not be generalized to childless samples. However, the ECR-R is increasingly being applied in research on parent-child relations and it is therefore becoming imperative that the factor structure should also be validated in parent samples and not only in childless individuals. Our study is the first to employ a sample of parents in a psychometric study. We must therefore emphasize that further studies within this area are required. Although our data do not support the expected two-factor model, we must caution against drawing conclusions regarding the best-fitting structure before further studies have assessed this matter. Finally, as only two previous studies have applied the ECR-R in a clinical population [10; 15], future research should further consider examining the psychometric properties of the ECR-R when data are derived from individuals with psychiatric difficulties, if it is to be valid for such groups.

The present study corroborates a previous study in a Norwegian sample, indicating that the models suggested by attachment theory do not provide optimal model fits. As in the previous study, a five-factor structure was found for Danish adults. However, our data do not support this conceptualization sufficiently at present, and further studies are required before firm conclusions can be fully drawn regarding Scandinavian samples. In particular as one cannot rule out that our sample may be limited by self-referral bias and thus not be generalizable to the overall population. Our findings, however, do highlight the importance of studies being conducted in multiple cultures since direct transferences from one culture to another may prove problematic.
